# Neuronal expression of Ca^2+^ oscillation initiator is linked to rapid neonatal growth in mice

**DOI:** 10.17912/micropub.biology.000325

**Published:** 2020-11-12

**Authors:** Woojin Kang, Kenji Yamatoya, Kenji Miyado, Mami Miyado, Yoshitaka Miyamoto

**Affiliations:** 1 Department of Reproductive Biology, National Research Institute for Child Health and Development, Setagaya, Tokyo 157-8535, Japan; 2 Institute for Environmental and Gender-Specific Medicine, Juntendo University Graduate School of Medicine, Urayasu, Chiba 279-0021, Japan; 3 Department of Molecular Endocrinology, National Research Institute for Child Health and Development, Setagaya, Tokyo 157-8535, Japan

**Figure 1. Neuronal extra-mitochondrial citrate synthase (eCs) expression and retarded neonatal growth in eCs-deficient (KO) male mice:**
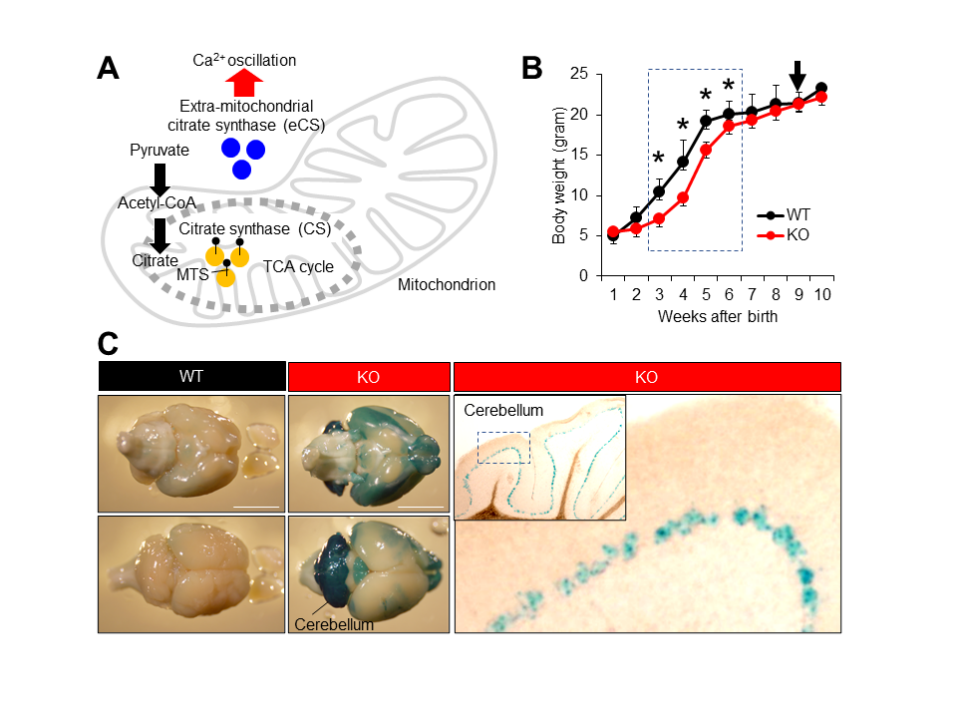
(A) Cooperative function of two citrate synthases, CS and eCS. (B) Growth curve of wild-type (WT) and *eCs*-KO mice. Ten mice from each genotype were weighed once a week. Dotted box: *eCs*-KO mice with significantly reduced weight. Arrow: *eCs*-KO mice brains highlighted with *LacZ* staining. Values are expressed as mean ± standard error of the mean. * *p* < 0.0001. (C) The *eCs* expression in adult mouse brains (9-week-old males). In the *eCs*-null allele, *eCs*-promoter drives the expression of the *LacZ* reporter gene. The *LacZ*-stained brain was viewed from dorsal (upper panel) and ventral (lower panel) surfaces from the same individual male. In the cerebellum, visualization of β-galactosidase activity in KO adult mice showed the *eCs* expression in a single cell layer lined inside the cerebellum, presumably the Purkinje cell layer. Dotted box in the inset: enlarged region. Scale bars: 5 mm.

## Description

Repetitive increases in the cytoplasmic calcium concentration (Ca^2+^ oscillation) control a wide variety of biological events (Dupont *et al.* 2011). In fertilization, a sperm-bearing factor, phospholipase C zeta 1 (PLCz1), triggers Ca^2+^ oscillation and resumes cell division in eggs. Citrate synthase (CS) is localized to the mitochondrial matrix, where it catalyzes a reaction between acetyl-coenzyme A (CoA) and oxaloacetate to form citric acid (Surpin and Chory 1997) (Figure 1A). The extra-mitochondrial form of CS (eCS) is encoded by a separate gene in mice and is expressed by alternative splicing from the *CS* gene in humans (Kang *et al.* 2020). eCS functions as a secondary factor triggering Ca^2+^ oscillation, which is transferred from the sperm to the eggs (Kang *et al.* 2020). More specifically, eCS has been found to trigger an initial Ca^2+^ spike using a PLCz1-independent mechanism. However, the role of eCS-triggered Ca^2+^ oscillation in broad cell functions is unknown.

Presently, we studied the neuronal involvement of eCS and its neuronal expression using *eCs*-deficient (KO) male mice carrying the *LacZ* reporter gene inserted into the *eCs*-null allele. KO mice were born healthy, but their body size was noticeably small. Therefore, KO mice were weighed daily after birth and compared with wild-type (WT) mice (Figure 1B). From the first to the second week, the body weight was comparable between KO and WT mice. However, KO mice continuously weighed less than WT mice during the third week (6.7 ± 0.5 g vs. 11.2 ± 1.1 g; *p* < 0.0001), fourth week (9.1 ± 0.4 g vs. 14.0 ± 2.5 g; *p* < 0.0001), fifth week (15.6 ± 0.6 g vs. 19.5 ± 0.6 g; *p* < 0.0001), and sixth week (18.3 ± 0.6 g vs. 20.4 ± 0.7 g; *p* < 0.0001). No difference was noted from the seventh week onward. Moreover, *eCS* expression was detected in a narrow layer of the cerebellar cortex, probably the Purkinje layer (Figure 1C). From this result, we assumed that *eCs* and *eCS*-expressed neuronal cells could regulate the rapid increase in body weight during childhood.

Growth retardation is linked to low concentrations of growth hormone (GH) in humans and mice. GH, also known as somatotropin, is a peptide hormone that mainly functions in growth, cell replication, and cell regeneration (Velloso 2008). GH stimulates the production of the insulin-like growth factor-1 (IGF-1) (also called growth-promoting hormone) to regulate overall body growth (Yakar *et al.* 2002). Moreover, GH release conclusively depends on changes in intracellular Ca^2+^ concentration, indicating a critical role of intracellular Ca^2+^ as a mediator of GH function (Cuttler *et al.* 1992). Upon the development of the central nervous system, IGF-1 is abundantly expressed in neurons, especially in Purkinje cells, to promote cell survival in the cerebellum (Torres-Aleman *et al.* 1994; Chrysis *et al.* 2001), suggesting a possible contribution of eCS to neuronal survival.

Otherwise, spontaneous Ca^2+^ oscillation is induced in astrocytes both in vitro and in vivo (Zhou *et al.* 2020). Ca^2+^ oscillation also controls the fate determination of cultured neural stem cells (Glaser *et al.* 2020). From our results, we concluded that *eCS* expressed in the Purkinje cell layer could trigger neuronal signaling via Ca^2+^ oscillation, subsequently enhancing the rapid growth observed during childhood.

Citrate has been shown to be a regulator of various biological processes, such as insulin secretion (Iacobazzi and Infantino 2014). Citrates act as suppressors of problematic events, such as in the reduction of oxidative stress and inflammation (Iacobazzi and Infantino 2014) and in the protection against traumatic brain injury (Kilbaugh *et al.* 2015). However, while it is known that citrates are specifically synthesized and released from astrocytes (Westergaard *et al.* 2017), presently, knowledge of the role of citrates is insufficient. Our results largely contribute to the understanding of eCS-mediated Ca^2+^ oscillation in the brain.

## Methods

**Animals**

Mutant mice were generated from C57BL/6-derived embryonic stem cell clones by injection into blastocysts from C57BL/6 mouse with a genetically deleted *Csl* (*eCs*) (Csl^tm1(KOMP)Vlcg^; ID14519) obtained from the Knockout Mouse Project (KOMP) repository (an NCRR-NIH-supported strain suppository; www.komp.org). Homozygous mice (C57BL/6 genetic background) were generated by subsequent intercrosses of heterozygous animals. For *LacZ* staining, 8-week-old male C57BL/6J mice were purchased from Japan SLC Inc. (Shizuoka, Japan) and their brains were used as control.

All mice were housed under specific, controlled pathogen-free conditions. Food and water were available ad libitum. All animal experiments were approved by The Institutional Animal Care and Use Committee of the National Research Institute for Child Health and Development (Experimental number, A2004-004).

***LacZ* staining**

After fixation with 4% paraformaldehyde (Wako Pure Chemical Industries, Osaka, Japan), mouse brains were washed three times with 0.1 M phosphate-buffered saline (pH 7.4) for 5 min. They were then incubated in β-gal staining solution [1 mg/mL 5-Bromo-4-chloro-3-indolyl β-D-galactopyranoside, 2 mM MgCl_2_, 5 mM potassium hexacyanoferrate (III), and 5 mM potassium hexacyanoferrate (II) trihydrate, 0.01% (w/v) sodium deoxycholate, 0.02 (w/v) NP-40] at 37 °C for overnight. To observe the *eCs* expression patterns, samples were embedded in Tissue-Tek OCT compound (Sakura, Finetek, Tokyo, Japan), frozen in liquid nitrogen, and cut into thin sections (10 mm) using a cryostat (CryoStar NX70, Thermo Fisher Scientific, Inc., MA).

**Statistical analysis**

Comparisons were made using one-way analysis of variance following Scheffe’s method, Mann–Whitney *U*-test, or Fisher’s exact test. Statistical significance was defined as *p* < 0.05. Results are expressed as the mean ± standard error of the mean.
